# Early Detection of Deep Venous Thrombosis in Trauma Patients

**DOI:** 10.7759/cureus.9370

**Published:** 2020-07-24

**Authors:** Stanton Nielsen, David O'Connor, Sanjeev Kaul, Jyoti Sharma, Massimo Napolitano, Gregory Simonian, Melissa Blatt, Tania Zielonka, Themba Nyirenda, Stephen Cohn

**Affiliations:** 1 Surgery, Hackensack University Medical Center, Hackensack, USA; 2 Statistics, Hackensack University Medical Center, Hackensack, USA

**Keywords:** venous duplex ultrasound, deep venous thrombosis, trauma, venous thromboembolism

## Abstract

Background

This study was performed to determine whether trauma patients are at an increased risk of developing deep venous thrombosis (DVT) within the first 48 hours of hospitalization.

Materials and methods

A retrospective review was performed using a prospectively maintained database of patients admitted to a trauma center during a five-year time period. Patients hospitalized for greater than 48 hours who received a screening venous duplex for DVT were included in the study.

Results

There were 1067 venous duplex scans obtained, 689 (64.5%) within the first 48 hours of admission (early DVT group), 378 (35.4%) after the first 48 hours (late DVT group). Only 142 (13.2%) patients had a positive duplex scan for DVT, 55 (early group), 87 (late group). Comorbid conditions of congestive heart failure (P = 0.02), pelvic fractures (P = 0.04), and a lower initial systolic blood pressure on presentation (p = 0.04) were associated with early DVT. Head trauma (P < 0.01), mechanical ventilation (P < 0.001), and transfusion of blood products (P < 0.001), were predictors of DVT in the late group.

Conclusions

Trauma patients are at an increased risk of developing venous thrombosis early in the hospital course due to comorbidities associated with trauma. Whereas, venous thrombosis in trauma patients diagnosed after the first 48 hours of hospitalization appears to be associated with prolonged patient immobility.

## Introduction

Venous thromboembolism (VTE) is a significant cause of morbidity and mortality in trauma patients and pulmonary embolism represents one of the most common causes of death in those surviving the first 24 hours of hospitalization [1]. Literature suggests the incidence of VTE in high-risk trauma patients without prophylaxis is 14% to 59% with rates of 3.6% to 33% for those patients on prophylaxis [2-5]. Traditionally, only those patients clinically suspected of having deep venous thrombosis (DVT) underwent venous duplex ultrasound (VDU) imaging. However, studies have shown that the majority of DVTs experienced by trauma patients are asymptomatic [6]. In an attempt to reduce the clinical sequelae of VTE in the trauma population, many centers now perform screening VDU on all high-risk patients and initiate prophylactic measures with chemoprophylaxis and intermittent pneumatic compression devices to reduce DVT [7-9]. There remains a lack of consensus amongst trauma surgeons on the factors qualifying a patient as high-risk, and therefore warranting surveillance VDU [10].

Classically described in Virchow’s triad, three major factors contribute to the pathogenesis of VTE: venous stasis, hypercoagulability and endothelial injury. Factors found to place trauma patients at moderate to high-risk for VTE have historically included the following: blunt mechanism of injury, spinal cord injury, long-bone or pelvic fractures, major head injury, severe vascular endothelial damage, the need for transfusion of blood products, advanced age, an injury severity score (ISS) greater than 15 and hypotension [2,8,11,12]. While screening VDU imaging of high-risk trauma patients remains a controversial practice, employing this modality naturally leads to an increase in the number of DVTs diagnosed [13-15].

There is little data focusing on the timing of diagnosis of DVT following injury. However, Azarbal et al. found that 30% of lower extremity DVTs were identified within the first week of admission and Hamada et al. found the median time of detection of DVT on serial screenings to be six days [16,17]. The aim of our study was to describe our experience with the early detection of DVTs in trauma patients. We postulated that trauma victims were at an increased risk of developing DVTs early in their hospital course.

## Materials and methods

This study is a retrospective review of a prospectively maintained trauma database at a level 2 trauma center. From May 2010 to March 2015, patients who were ≥18 years of age, hospitalized for at least 48 hours and received a screening venous duplex for deep vein thrombosis were included in the study. Evaluation of high-risk trauma patients with VDU was left to the discretion of the trauma surgeon. Patients were further characterized into those that had a positive screening duplex ultrasound for a DVT and those with negative screenings for DVT within 30 days of admission. Patients were excluded from the study if they had a prior history of DVT. Patients were further defined into groups based on the timing of VDU screening: early, within the first 48 hours of hospitalization, or late, after 48 hours. DVTs were classified based on location as above the knee (AK) or below the knee (BK). This study was approved by the institutional review board of Hackensack Meridian Health.

Baseline patient demographics were obtained including: age, gender, body mass index (BMI), Glasgow Coma Scale (GCS), injury severity score (ISS), co-morbidities, and abbreviated injury score (AIS). Outcome variables included total length of ventilator days, intensive care unit (ICU) and hospital length of stay (LOS), pulmonary embolism, major diagnoses/injuries, inferior vena cava (IVC) filter placement, blood transfusions, and mortality. VTE prophylaxis consisted of pharmacologic and/or mechanical methods unless contraindicated. Pharmacologic prophylaxis was comprised of enoxaparin or heparin, unless the patient was on therapeutic anticoagulation for another diagnosis.

Data analysis was performed using SPSS software, version 23 (IBM Corp., Armonk, NY), for Windows. A comparison between early and late development of DVT was performed using a Mann-Whitney U test or chi-square tests. Data was then analyzed using multivariate regression, with a p-value < 0.05 being statistically significant.

## Results

From May 2010 to March 2015, 1067 of the 4147 patients that were admitted to our trauma center were determined by the surgical team to be moderate to high-risk for DVT. These individuals underwent VDU screening (Table 1). The overall rate of DVT found in the population screened with VDU was 13.3% (N = 142). Of the 1067 venous ultrasounds obtained, early screening (≤48 hrs of admission) made up a majority of the patients screened, 689 (64.5%).

**Table 1:** Trauma Patients With & Without VDU Screening Values are n (%) for all except where stated. GCS: Glasgow Coma Scale; VDU: Venous Duplex Ultrasound; SD: Standard Deviation; Hemo: Hemothorax; Pneumo: Pneumothorax.

Characteristic	No Duplex, N = 3,080	Duplex, N = 1,067
Gender (% male)	1834 (59)	659 (61)
Age (mean ± SD, years)	57 ± 22	61 ± 21
Body Mass Index	26.6 ± 5.45	27.5 ± 5.8
Intubated	249 (8)	228 (21)
Ventilator Days (mean ± SD)	5.9 ± 8.6	12 ± 18
GCS Admission (median ± SD)	14 ± 2.8	13 ± 4
Injury Severity Score	9 ± 8	13 ± 11
Intensive Care Unit Admission	704 (22)	498 (47)
Length of Stay (mean ± SD, days)	6 ± 13	13 ± 23
Traumatic Brain Injury	733 (24)	328 (31)
Pelvic/Femur Fracture	215 (7)	202 (19)
Vertebral Fracture	361 (12)	32 (3)
Multiple Ribs/Hemo/Pneumo	218 (7)	100 (9)
Other Fracture	679 (22)	209 (20)
Concussion/Contusion	605 (19.6)	196 (18)
Pre-admission Coumadin	170 (5.5)	98 (9)
Pre-admission Aspirin or Plavix	423 (13.7)	173 (16)
History of Coronary Artery Disease	217 (7)	77 (7)
History of Hypertension	1171 (38)	469 (44)
History of Stroke	88 (23)	39 (4)
History of Diabetes	358 (12)	147 (14)
Inferior Vena Cava Filter Placement	3 (0.1)	62 (6)
Pulmonary Embolism	4 (0.13)	22 (2)
Alive at Discharge	2986 (97)	1010 (95)

In the early screening group, a total of 55 of the 689 patients (8%) were found to have a DVT. Only 20 (36%) of the 55 early DVTs were located above-knee, whereas 35 (64%) were below-knee DVTs. All above-knee DVT patients were treated; 13 patients underwent placement of an inferior vena cava (IVC) filter, while the remaining seven patients received therapeutic anticoagulation. Five of the early screening group had a co-existing pulmonary embolism at the time of DVT diagnosis.

Twenty-six variables were evaluated as potential risk factors for development of a DVT in our trauma population using a multivariate analysis (Table 2). Among the patients who underwent early screening VDU, a positive finding of DVT was associated with the diagnosis of hypotension on admission (p = 0.04), the presence of a pelvic fracture (P = 0.04), and pre-existing congestive heart failure (P = 0.02).

**Table 2:** Associations Between Factors & Occurrence of Early DVT DVT: Deep venous thrombosis

Characteristic	Odds Ratio (95% Confidence Interval)	P-value
Age (years)	1.01 (0.99 – 1.02)	0.50
Gender, male	0.86 (0.48 – 1.52)	0.59
Body Mass Index	1.03 (0.98 – 1.07)	0.25
Systolic Blood Pressure	0.99 (0.98 – 1.00)	0.04
Glasgow Coma Score	0.98 (0.90 – 1.06)	0.55
Intubated	0.90 (0.44 – 1.85)	0.78
Admission to Intensive Care Unit	1.32 (0.76 – 2.29)	0.33
Intensive Care Unit Duration	1.04 (0.99 – 1.09)	0.15
Total Ventilator Days	1.02 (0.99 – 1.05)	0.14
Injury Severity Score	1.01 (1.00 – 1.04)	0.25
Trauma and Injury Severity Score	0.41 (0.06 – 2.79)	0.36
Red Blood Cell Transfusion	1.51 (0.80 – 2.86)	0.21
Fresh Frozen Plasma Transfusion	0.93 (0.38 – 2.24)	0.87
Platelet Transfusion	0.64 (0.26 – 1.71)	0.40
Traumatic Brain Injury	0.67 (0.35 – 1.28)	0.23
Mechanism of Injury		
Fall	0.85 (0.49 – 1.47)	0.56
Motor Vehicle Accident	1.17 (0.86 – 1.57)	0.32
Other	0.88 (0.43 – 1.79)	0.72
Abbreviated Injury Scale:		
Head ≥2	0.73 (0.38 – 1.42)	0.36
Chest ≥2	1.40 (0.68 – 2.88)	0.36
Pelvic Girdle ≥2	1.89 (1.02 – 3.50)	0.04
Lower extremity	0.75 (0.26 – 2.15)	0.59
History of:		
Coronary Artery Disease	1.40 (0.57 – 3.43)	0.46
Congestive Heart Failure	0.35 (1.21 – 6.24)	0.02
Myocardial Infarction	1.77 (0.51 – 6.16)	0.37
Hypertension	0.77 (0.44 – 1.34)	0.36

Of the 379 patients who received late VDU screening (>48 hrs. after admission), 87 (23%) were found to have a DVT. The DVT was located above-knee in the majority of patients (N = 48, 55%). Risk factors for late DVT included: traumatic brain injury (P < 0.05), craniotomy (P < 0.01), mechanical ventilation (P < 0.001), and blood, plasma or platelet transfusion (all P < 0.001).

## Discussion

The principal findings of our study include the following. Only 6.4% of our trauma population classified as moderate to high-risk for DVT and had an above-knee DVT. Eight percent of patients screened for early DVT had positive findings. Risk factors associated with early DVT (DVT occurring within the first 48 hours) were admission hypotension (p = 0.04), the presence of pelvic fractures (P = 0.04), and pre-existing congestive heart failure (P = 0.02). Patients screened for late DVT (DVT identified more than 48 hours after admission) found positive findings in 23%. Predictors of late DVT included: traumatic brain injury (P < 0.05), mechanical ventilation (P < 0.001), and blood, plasma or platelet transfusion (all P < 0.001).

The predictors of venous thrombosis in our trauma patients were consistent with risk factors found in other studies. Prior investigations identified major head injury, spinal cord injury, long-bone and pelvic fractures, hypotension, advanced age, the transfusion of blood products, and an injury severity score greater than 15 all as risk factors [2,11,12,18]. Supporters of surveillance VDU in high-risk trauma patients argue earlier diagnosis of DVT results in earlier treatment, possibly preventing propagation of the DVT and development of a pulmonary embolism [10,13,16]. However, other studies show screening for DVT in trauma patients to have little effect in preventing pulmonary emboli and suggest that surveillance studies are not cost-effective [19,20].

In our investigation, patients who had hypotension on initial presentation, those who sustained pelvic fractures, and patients with known congestive heart failure appeared to be at an increased risk of venous thrombosis in the first 48 hours. Similarly, Hamada et al. found that patients with DVT had associated risk factors of hypotension during initial management along with those sustaining pelvic fractures, although timing of DVT with these specific risk factors was not stated [17]. Additional studies have also demonstrated pelvic fractures predisposing trauma patients to DVT and PE [8,14,21-23]. Stannard et al. showed a 12.2% incidence of DVT in their patients sustaining pelvic trauma [21]. The literature has also shown an association with DVT in patients with known congestive heart failure (CHF) [24]. Although prior to this study, there was a paucity of data supporting the notion that trauma patients with known CHF are at increased risk of DVT.

For those identified with DVT later in their hospital course, our study demonstrated statistically significant risk factors which include head trauma, diagnosis of traumatic brain injury, mechanical ventilation, and the need for transfusion of blood products. Similarly, others have shown patients experiencing head trauma and mechanical ventilation are at high-risk for DVT [7,10,11]. There is also a well-known association between transfusion of blood products and development of DVT [2,12,14]. Our study data further differentiated that trauma patients undergoing transfusion of red blood cells (RBC), fresh frozen plasma (FFP), and platelets, each individually had a statistically significant increased risk of late DVT formation.

This study had limitations; our data was collected retrospectively and at a single institution. Our decision to screen with VDU was left to the discretion of the trauma surgeon, without standardized criteria qualifying the patients as low, moderate, or high-risk. Additionally, a large majority of our population who had late VDU screening did not undergo screening within the first 48 hours of admission and could not be ruled out as having an early DVT.

## Conclusions

The early post-admission screening of trauma patients with duplex ultrasound for venous thrombosis is supported by our study. A subset of injured patients appears to be at particularly increased risk of developing DVT within the first 48 hours of admission, specifically those with hypotension on initial presentation in the trauma bay, patients sustaining pelvic fractures, and those with preexisting congestive heart failure. Larger prospective studies are needed to establish which other patient groups are at high-risk for the development of deep vein thrombosis shortly after hospital admission and may therefore benefit from prompt surveillance.
